# Deciphering the true antiproliferative target of an MK2 activation inhibitor in glioblastoma

**DOI:** 10.1038/cddis.2015.384

**Published:** 2016-01-28

**Authors:** P E Brennan

**Affiliations:** 1 Structural Genomics Consortium, Target Discovery Institute, Nuffield Department of Medicine, University of Oxford, Oxford, UK

There has been much interest in developing inhibitors of the checkpoint kinases Chk1/2 to augment the effects of DNA-damaging agents for chemotherapy.^1^ In addition to Chk1/2, Wee1 and MAPK-activated protein kinase-2 (MK2) have emerged as additional key regulators of cell-cycle checkpoints.^2^ Evidence has accumulated that implicates MK2 as a target for chemo-sensitization in both p53-proficient and deficient tumors.^3^ MK2 is an attractive target for cancer treatment as MK2 inhibition has the potential to regulate the cell-cycle effects of the p38–MAPK pathway without inhibition of poly-functional p38 itself, which regulates many cellular signaling networks. MK2 inhibition in the absence of synergistic chemotherapy had not been investigated for its inherent cytotoxicity, and Munoz published a study aimed to fill this gap in our knowledge of MK2.^4^ For their MK2 cytotoxicity study in glioblastoma cells, Munoz *et al.* combined the use of siRNA and chemical probes. Three MK2 inhibitors were chosen for their distinct structures and mechanism of action (Figure 1a). CMPD1 is a non-ATP competitive inhibitor, which prevents phosphorylation and activation of MK2 via binding to p38–MAPK (*K*_i_ 330 nM).^5^ CMPD1 is not simply a non-selective p38 inhibitor as it shows no inhibition of the phosphorylation of two other p38 substrates, ATF2 and MBP. MK2i is a classical ATP-competitive kinase inhibitor, which inhibits MK2 (IC_50_ 126 nM).^2^ PF-3644022 is a more potent ATP-competitive MK2 inhibitor (*K*_i_ 5 nM).^6^ Although MK2i and PF-3644022 are both ATP-competitive MK2 inhibitors, they differ significantly in potency and are structurally orthogonal.^7^

When glioblastoma cell lines with different p53 and EGFR backgrounds were treated with CMPD1, the expected decrease in cell proliferation for all cell types was observed at concentrations in line with the reported *K*_i_ of MK2 phosphorylation (Figure 1a). Surprisingly, when the more potent, direct MK2 inhibitors MK2i and PF-3644022 were used, little effect was seen on proliferation. When used at much higher concentrations, antiproliferative effects were eventually seen but the EC_50_'s were at concentrations 200–10 000 times higher than the *in vitro* inhibition. siRNA-KD of MK2 showed effects similar to those of the direct MK2 inhibitors: at 90% KD, proliferation decreased by only 15%. The combination of CMPD1 and MK2 KD showed no change compared to CMPD1 treatment alone, indicating that the effects were independent.

These experiments suggested that CMPD1 may not be exerting its antiproliferative effects through inhibition of MK2 signaling. Rather than ignoring these conflicting results that did not support their hypothesis, Munoz *et al.* chose to decipher the role of CMPD1 in preventing glioblastoma proliferation and discovered an additional molecular target.

Examination of the effect of CMPD1 at higher concentrations showed an increase in MK2 phosphorylation indicative of the cellular stress response. Such an effect is consistent with cell-cycle arrest, and a number of markers were examined to confirm that upon CMPD1 treatment U87 cells showed an increase in G2/M followed by an increase in the SubG1 population (Figure 1b).

Following arrest, CMPD1-treated glioblastoma cells entered apoptosis as indicated by increase in annexin-V and cleaved-PARP1 (cPARP1). A decrease in Bcl-X_L_ via proteasomal degradation and Mcl-1 levels mechanistically linked the cell-cycle arrest with the induction of apoptosis (Figure 1c).

The final clue to how CMPD1 was exerting its cytotoxic effects came from examining U87-cell morphology following compound treatment (Figure 1d). The cells showed changes in the cytoskeleton upon staining for tubulin. The loss of a well-formed mitotic spindle and formation of multinuclear cells was similar to what is seen upon treatment with the tubulin polymerization inhibitor vinblastine. *In vitro* fluorescent detection of tubulin polymerization showed that CMPD1 was indeed a potent inhibitor of this process. Munoz *et al.* completed their study by showing that CMPD1 is less cytotoxic to normal astrocytes over glioblastoma cells.

The study by Munoz is an example of deciphering the target of a small-molecule inhibitor using thorough cell biology techniques. This work is more significant due to the reported role of CMPD1 as an MK2 inhibitor, which is irrelevant to its antiproliferative effects in glioblastoma.

In early kinase drug discovery, it was common to discover additional kinase targets of putative selective inhibitors. In response to this incomplete characterization of inhibitors, the number of kinases available for selectivity screening increased dramatically to cover the majority of the kinome. It is increasingly common to have the complete kinome selectivity of an inhibitor disclosed.^8^ In addition to kinase off-targets, there have been a number of kinase inhibitors that have recently been disclosed to have other pharmacology beyond kinases. For example, the c-Met inhibitor tivantinib is also reported to have potent anti-tubulin activity much like CMPD1.^9^ The off-target activity of kinase inhibitors has also strayed into bromodomains.^10^

As chemical probes are used more in target discovery to link a phenotype to a target via small-molecule inhibition just as Munoz was attempting to do with CMPD1, it is imperative that we understand the pharmacology of the tools used. Technologies such as kinobeads and ActiveX attempt to do this by interrogating the binding of a molecule to the entire active kinome in a cell lysate, but are still limited to one protein family.^11^ CETSA potentially extends the biological annotation of a chemical probe to the entire proteome, but in practice is limited to proteins sensitive enough to stabilization by ligand binding.^12^ Future approaches to chemical probe characterization will likely include pharmacological finger-print matching via transcriptomics and high-content imaging.^13^

A final, low-cost way to increase the utility of chemical probes is via better sharing of pharmacology annotation. Munoz put considerable effort into deciphering the true target of CMPD1 in preventing proliferation. The anti-tubulin effects of CMPD1 should be immediately known to the next researcher who purchases this compound as an MK2 inhibitor. Although commercial vendors have been essential for making chemical probes available for target discovery, they can be slow in responding to further characterization of probes in the literature. Although tivantinib was first described as a tubulin inhibitor in February 2013,^9^ of the 22 commercial suppliers of tivantinib listed in eMolecules and ChemSpider, only Cayman Chemical mentions the anti-tubulin activity in the product description (eMolecules (www.emolecules.com) and ChemSpider (www.chemspider.com) was searched on 20 September 2015 for ‘tivantinib.' The catalog of each of the 27 suppliers listed in either search were subsequently searched for ‘tivantinib' or ‘ARQ-197' and it was found in 22 supplier catalogs. Nine suppliers have no description for tivantinib beyond the structure and molecular weight. Twelve list it as a selective c-Met inhibitor. Only Cayman Chemical describes tivantinib's additional anti-tubulin activity). The remaining suppliers' catalogs either lack biological annotation for tivantinib or erroneously describe it as a selective c-Met inhibitor.

The lack of consistent descriptions of an individual chemical probe's strengths and weaknesses, including poly-pharmacology, was the subject of a recent letter from Arrowsmith *et al*,^14^ who advocated the creation of an easily accessible information source for researchers to add their own references and findings for chemical probes. Since then, the website www.chemicalprobes.org has been set up, which aims to create a first point-of-call for those interested in finding chemical probes with the latest annotation and using them in their research. Initiatives like this should ensure that the hard work done by Munoz *et al.* characterizing the true pharmacology of CMPD1 is not missed.

## Figures and Tables

**Figure 1:**
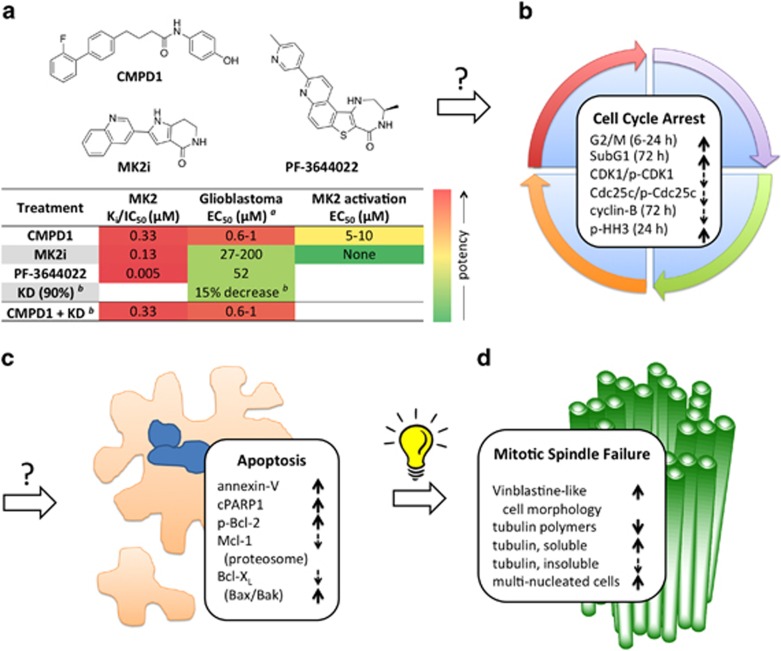
Deciphering the role of CMPD1 in glioblastoma. (**a**) Effect of chemical probes and siRNA knockdown (KD). (**b**) CMPD1 caused profound changes to the cell cycle. (**c**) Following arrest, cells entered apoptosis. (**d**) Morphology and tubulin state are from mitotic spindle disruption. *^a^*Cells: U87 (WT-p53), U87-EGFRvIII (constitutively-active EGFRvIII), A172 (mutant-p53), U251 (mutant-p53); *^b^*siRNA-KD: 90% decrease in MK2-protein and 15% decrease in U87 proliferation
